# Plasma 25-Hydroxyvitamin D Concentrations and Serum and Salivary C-Reactive Protein in the Osteoporosis and Periodontal Disease Study

**DOI:** 10.3390/nu13041148

**Published:** 2021-03-31

**Authors:** Sarah E. Twardowski, Jean Wactawski-Wende, Kathleen M. Hovey, Christopher A. Andrews, Hailey R. Banack, Michael J. LaMonte, Amy E. Millen

**Affiliations:** 1 Department of Epidemiology and Environmental Health, School of Public Health and Health Professions, University at Buffalo, State University of New York, Buffalo, NY 14214, USA; sarah.twardowski@mail.mcgill.ca (S.E.T.); jww@buffalo.edu (J.W.-W.); koreilly@buffalo.edu (K.M.H.); hrbanack@buffalo.edu (H.R.B.); mlamonte@buffalo.edu (M.J.L.); 2 Department of Ophthalmology and Visual Sciences, University of Michigan, Ann Arbor, MI 48105, USA; candrews@buffalo.edu

**Keywords:** vitamin D, C-reactive protein, saliva, oral inflammation, periodontal diseases, epidemiology

## Abstract

Vitamin D has been hypothesized to play an important role in preventing the development and progression of periodontal disease, but the underlying immune modulatory mechanisms remain understudied. We examined the cross-sectional association between biomarkers of vitamin D status and C-reactive protein (CRP) among postmenopausal women aged 53–81 years. Linear regression was used to examine the association between plasma 25-hydroxyvitamin D (25[OH]D) concentrations, a biomarker of vitamin D status, and both salivary and serum CRP concentrations in 567 women from the Buffalo Osteoporosis and Periodontal Disease (OsteoPerio) Study (1997–2000). CRP concentrations were measured with multiplex arrays and transformed for normality using the natural log. Concentrations above and below the limit of detection were included in analysis as right- and left-censored observations. An inverse association was observed between 25(OH)D and salivary CRP in a model adjusted for age, smoking status, frequency of tooth brushing and flossing, and hormone therapy use (−7.56% difference in salivary CRP concentrations per 10 nmol/L increase in 25(OH)D, 95% CI: −12.78 to −2.03). Further adjustment for percent body fat attenuated this association (−2.48%, 95% CI: −7.88 to 3.24). No significant associations were found between 25(OH)D and serum CRP. Plasma vitamin D concentrations were not associated with salivary or serum CRP concentrations in this cohort of postmenopausal women.

## 1. Introduction

Periodontal disease is an inflammatory condition affecting nearly half of US adults over age 30 [1]. It is initiated by the accumulation of pathogenic oral bacteria that induce an immunopathologic response by the host, leading to tissue destruction [2]. Previous literature has hypothesized that vitamin D may play an important role in preventing the development and progression of periodontal disease [3,4,5,6,7,8]. In support of this proposed relation, we previously observed an inverse, cross-sectional association between vitamin D status, assessed with plasma 25-hydroxyvitamin D (25[OH]D) concentrations, and acute gingival bleeding among postmenopausal women [4]. We further hypothesized that this association may be explained, in part, by the anti-inflammatory properties of vitamin D [9]. Support for this hypothesis is provided by results of in vitro studies that have shown the active vitamin D hormone, 1,25-dihydroxyvitamin D (1,25[OH]_2_D), mediates bacterial infections and modulates immune responses, providing evidence that it could potentially mitigate oral inflammation [9,10,11].

To better understand our observed association between 25(OH)D and acute gingival bleeding in our cohort of postmenopausal women, we examined associations between plasma 25(OH)D concentrations and both salivary C-reactive protein (CRP) and serum CRP concentrations, acute measures of inflammation. Previous research has shown that the correlations between salivary and serum CRP are moderate [12]. Positive associations have been observed between plasma CRP measures and presence of periodontal disease [13], and it is thought that biomarkers of oral inflammation, as seen with periodontal infections, can spill into the systemic circulation and contribute to the circulating pool of serum inflammatory markers [14]. To the best of our knowledge, only a few studies have investigated associations between salivary CRP concentrations and periodontal disease. A short-term study of experimentally induced gingivitis in eight women observed no association between salivary CRP concentrations and gingivitis [15]. A different study observed higher concentrations of salivary CRP in participants with chronic periodontitis compared to healthy controls and may suggest that salivary CRP indicates increased inflammation in the oral cavity due to local disease [16]. Furthermore, limited research has investigated associations between blood concentrations of 25(OH)D and biomarkers of inflammation within the oral cavity, and this research has not appeared to include measures of salivary CRP [17,18,19,20]. For our analyses, we hypothesized that 25(OH)D concentrations would be inversely associated with serum CRP, as previously observed in other studies [21,22,23,24,25,26,27], and that 25(OH)D concentrations would also be inversely associated with salivary CRP concentrations.

## 2. Materials and Methods

### 2.1. Study Design and Sample

We used baseline data from postmenopausal women in the Buffalo Osteoporosis and Periodontal Disease (OsteoPerio) study, an ancillary study of the Women’s Health Initiative Observation Study (WHI OS). The WHI OS is a prospective study established to assess factors associated with the morbidity and mortality among postmenopausal women [28]. Participants were followed annually by completing health update questionnaires and returning to the clinic for a follow-up clinic visit (1997–2000) 3 years after their baseline visit.

Prior to the 3-year follow-up visit, WHI participants at the Buffalo Clinical Center (*n* = 2249) were invited by mail to participate in the OsteoPerio Study. The OsteoPerio study was established to examine the association between osteoporosis, oral bone loss, periodontal disease, and systemic health outcomes. Women were excluded from participation if they had <6 teeth, a history of bone disease other than osteoporosis, bilateral hip replacement, a cancer diagnosis in the past 10 years, serious illness, or recent oral X-rays or a florescent dye test (temporary ineligibility). In total, 1362 participants enrolled in the OsteoPerio study [29].

After completion of clinical and oral examinations, an additional 21 women were excluded from the sample due to missing oral radiographs (*n* = 16) and missing questionnaire forms (*n* = 5). Because funding for blood collection was not available until later in the study, 407 of the remaining 1341 participants were missing fasting blood samples. One woman was excluded from our analyses because her reported 25(OH)D value exceeded biologic plausibility (530 nmol/L), leaving 933 women with measured 25(OH)D concentrations. Saliva samples were analyzed only if they were collected on the same day as the blood draw. As a result, there were fewer saliva samples than serum (839 serum CRP and 613 had salivary CRP), and only 577 participants had measures for both. Women were further excluded from the final analysis if they were missing frequency of flossing (*n* = 1), percent body fat (%BF) (*n* = 5), and the gingival bleeding on probing (BOP) measurement (*n* = 4). This left an analytic sample of *n* = 567 with all data available (Supplemental Figure S1). All study participants signed informed consent and the study protocol was approved by the University at Buffalo’s Health Sciences Institutional Review Board.

### 2.2. Plasma 25(OH)D

Fasting blood samples were collected by venipuncture during the clinic visit, prior to the dental exam, and processed using a standardized protocol. Samples were taken to the lab and processed within 90 min of the blood draw. Samples were processed, and both plasma and serum samples were aliquoted into 0.5 mL cryogenic straws, placed in −80 °C freezers for 24 h, and then submerged into liquid nitrogen at −196 °C for long-term storage. As previously described [30], plasma 25(OH)D concentrations was measured in samples by competitive chemiluminescence immunoassay (DiaSorin LIAISON 25(OH)D assay). The within-pair coefficient of variation (%CV) was 4.9%, determined by blinded duplicate quality control samples nested into each batch [30].

### 2.3. Salivary and Serum CRP

Fasting whole saliva samples (5 mL) were collected from participants at the clinic exam prior to the blood draw and dental examination. Participants with difficulty producing enough saliva were offered the option to chew a sterile rubber band to help stimulate saliva production. All other samples were unstimulated. Samples were transferred to 0.5 mL cryogenic straws which were sealed and stored in −80 °C freezers for 24 h before being submerged in liquid nitrogen (−196 °C) [31]. Serum from blood was collected and stored as described above. Cryogenic straws of saliva and serum were retrieved from liquid nitrogen and shipped on dry ice to the Forsyth Institute (Boston, MA) and remained in −80 °C until analysis. CRP concentrations were measured in serum and saliva samples to determine whether serum and saliva inflammatory measures correlate and to determine associations of the biomarkers with clinical measures of periodontal disease and bone density measures [31]. Samples were sent to the Forsyth laboratory blinded to health outcome and personal information. Samples were thawed on the day of analysis and analyzed using multiplex sandwich immunoassay powered by Luminex xMAP technology on a 100 Bio-Plex Platform (as previously described [31]). The CV for salivary CRP was 15.37% and serum CRP 20.65% were below the acceptable quality control %CV for blind duplicates defined as <20% and <25%, respectively [31,32]. Salivary CRP concentrations are quantified at lower levels than serum CRP. We therefore used the unit ng/mL for both serum and saliva (1000 ng/mL is equivalent to 1 mg/L).

### 2.4. Clinic Visit and Questionnaires

Participants completed questionnaires on family and personal health history, supplement use and current medications, daily life, physical activity, and oral health history and practices, as well as other demographic data (e.g., age, race, and education) [29]. All participants underwent a clinical oral examination from trained and calibrated dental examiners using standardized protocols modeled after the National Institute for Dental and Craniofacial Research (NIDCR) Criteria for Dental Examinations (as previously described [4,29,33]). During the clinic visit, participants had height and weight measured by trained staff (as previously described [34]). Participant %BF was measured directly using whole body dual X-ray absorptiometry (DXA) (QDR-4500A, Hologic, Bedford, MA, USA) scans [35].

### 2.5. Statistical Analyses

The distribution of participant characteristics was examined in relation to quartiles (Q) of plasma 25(OH)D concentrations and tertiles (T) of CRP. Quantile cut-off points were chosen based on univariate distribution of 25(OH)D and CRP, respectively. Periodontal disease related variables (pocket depth (PD), number of teeth present, and alveolar crestal height (ACH) and the Centers for Disease Control and Prevention/American Academy of Periodontology (CDC/AAP)-defined periodontal disease) were assessed and reported as descriptive data, but not included in main analyses.

To minimize the potential bias that can occur from excluding data outside the limits of detection (LOD), we treated salivary and serum CRP values above the upper LOD as right-censored values and values below the lower LOD as left-censored values. This was done using PROC LIFEREG in SAS version 9.4 (SAS Institute Inc., Cary, NC, USA). Upper and lower limits for the censored data were set as the adjusted minimum and maximum LOD values for each cytokine, respectively (salivary CRP 0.002, 15.88 ng/mL; serum CRP 12.94, 11,716.30 ng/mL [31]). CRP was modeled using a lognormal distribution, as previous literature suggests that CRP concentrations tend to be highly right skewed [36,37].

We regressed the natural logarithm-transformed continuous measure of saliva or serum CRP concentrations on the continuous measure of plasma 25(OH)D concentration. Plasma 25(OH)D concentrations were rescaled prior to regression, for clinical significance, by dividing by a constant so that 1 unit was equivalent to 10 nmol/L. Effect estimates (i.e., regression coefficient, β) and corresponding 95% confidence intervals (CIs) were back-transformed into percentage changes in CRP (and corresponding 95% CIs) using the following equation [38]:

$$\%\Delta y=100\times (e^{\beta_{1}}-1)$$

The resulting estimates were interpreted as percentage differences in CRP concentrations per 10 nmol/L increase of 25(OH)D, representing a comparison of 25(OH)D concentrations between 2 subjects whose CRP differed by 10 nmol/L.

We adjusted for the following covariates: age (years), hormone therapy usage (never/former or current use of estrogen and/or progestin), frequency of teeth brushing (less than twice, twice, more than twice per day), and frequency of flossing (not every week, once a week, 2 to 6 days/week, or every day). We also adjusted for adiposity, assessed as %BF, as adiposity is both a strong predictor of 25(OH)D [39] and CRP and thus a potential confounder of these associations. We report results of models with and without adjustment for %BF. We further stratified by %BF (<40% and ≥40% BF) according to cut-points previously defined for this cohort [35].

Further, sensitivity analyses were conducted to explore the effect of hypertension and cardiovascular disease (CVD) on the association between 25(OH)D and CRP. CRP is an acute phase reactant protein which increases in response to pathogenic invasion, but also remains elevated with chronic infections and inflammatory conditions such as CVD. Elevated serum CRP is a standard marker for CVD risk. Few studies have investigated whether salivary CRP holds the same validity as a marker of risk [40,41,42,43]. To investigate this, we repeated our analyses, excluding individuals diagnosed with hypertension and CVD.

## 3. Results

### 3.1. Sample Characteristics by Plasma 25(OH)D

In the current study, 35% of women at baseline had deficient or inadequate vitamin D status (25[OH]D < 50 nmol/L), as defined by the Institute of Medicine’s summary conclusions of vitamin D deficiency in relation to bone health [44]. Mean concentrations of plasma 25(OH)D in quartile groups ranged from 31.3 ± 8.5 nmol/L (Q1) to 87.7 ± 15.5 nmol/L (Q4), with an overall sample mean of 58.7 ± 9.4 nmol/L (Table 1). Women with low 25(OH)D (Q1) were, on average, slightly older, had greater %BF, had fewer teeth, and experienced a greater proportion BOP compared to women with high 25(OH)D (Q4). Women in Q1 compared to Q4 were less physically active, less likely to be current hormone therapy users, more likely to never visit the dentist or visit only with a problem, and reported less frequent tooth brushing and flossing. Of the sample, 4% were current smokers and 5% were diagnosed with diabetes.

### 3.2. Sample Characteristics by Salivary and Serum CRP

Salivary CRP concentrations within LOD (*n* = 553) ranged from 0.002 ng/mL to 15.9 ng/mL, with a median (interquartile range (IQR)) of 0.51 (1.05) (Supplemental Table S1). Thirteen observations were below LOD and one observation was above LOD. Women with low (T1) compared to high (T3) salivary CRP concentrations, on average, had less %BF, more teeth, and experienced a smaller proportion of BOP upon assessment. Women in T1 compared to T3 were less likely to be current hormone therapy users or diabetic and reported greater frequency of tooth brushing and flossing (Supplemental Table S1). Serum CRP concentrations within LOD (*n* = 501) ranged from 12.9 ng/mL to 11,716.3 ng/mL (median (IQR) = 2833.30 (3516.9)). Sixty-six observations were above LOD, and none were below LOD. Women in T1 compared to T3 for serum CRP concentrations, on average, had less %BF and were less likely to be current hormone therapy users.

The Pearson correlation and 95% CI between serum and salivary CRP were *r* = 0.60 (0.55, 0.65).

### 3.3. Association between Plasma 25(OH)D and Salivary and Serum CRP

The associations between CRP concentrations (salivary and serum) and concentrations of 25(OH)D are shown in Table 2 and Supplemental Figure S2a,b. After adjustment for age, hormone therapy usage, and frequency of tooth brushing and flossing, we observed an inverse association between salivary CRP (−7.56% change in salivary CRP concentrations per 10 nmol/L increase in 25(OH)D, 95% CI: −12.78 to −2.03), This association was attenuated after further adjustment for %BF (−2.48%, 95% CI: −7.88 to 3.24). No statistically significant findings were observed between plasma 25(OH)D concentrations and serum CRP measures.

In order to better understand the role of body fat in this association, we also examined to what extent the associations between 25(OH)D and both salivary and serum CRP were modified by %BF (Table 3) No associations were observed when stratifying by %BF for serum or salivary CRP.

To investigate whether the elevated CRP observed in our sample may be related to systemic conditions known to be associated with increased inflammation, we repeated our analyses, excluding individuals diagnosed with hypertension (*n* = 187) and CVD (*n* = 53). Results excluding these individuals were similar to original analyses (data not shown).

## 4. Discussion

We examined the cross-sectional association between plasma 25(OH)D and oral inflammation assessed with salivary CRP concentrations and systemic inflammation, assessed with serum CRP concentrations, in a cohort of postmenopausal women aged 53–81 years. After adjustment for age, hormone therapy usage, and frequency of tooth brushing and flossing, we observed a significant inverse association between plasma 25(OH)D and salivary CRP and an inverse, but not statistically significant, association with serum CRP. After adjustment for %BF, the association with salivary CRP was attenuated and no longer statistically significant. Our study suggests that plasma 25(OH)D concentrations are not associated with salivary or serum CRP concentrations.

CRP is a non-specific marker of inflammation, and few studies have assessed the role of salivary CRP independent of CVD risk [40,41,42,43]. Vitamin D is thought to have anti-inflammatory properties [9,10,11], and some studies have observed that higher concentrations of 25(OH)D are associated with lower concentrations of systemic CRP [21,22,23,24,25]. However, few studies have investigated the relationship between 25(OH)D and salivary CRP, specifically in humans. Our study helps fills this gap in our understanding of the relationship between vitamin D status and oral inflammation.

Because we observed inverse associations between 25(OH)D and acute gingival bleeding in this cohort [4], we hypothesized that we would also observe an inverse association between biomarkers of inflammation (serum and salivary CRP) and plasma 25(OH)D if vitamin influenced gingival bleeding through an inflammatory pathway. In vivo research supports that the active hormone of vitamin D, 1,25-dihydroxyvitamin D, suppresses cytokine production in gingival fibroblasts [45] and *Porphyromonas gingivalis-*stimulated macrophages [46]. A recent clinical study in 42 adults (21 healthy controls and 21 patients with periodontal disease aged 25 to 60 years) recruited from a dental clinic examined correlations between different cytokines and both serum and salivary concentrations of 25(OH)D [17]. They did not measure CRP but did observe negative correlations between salivary 25(OH)D and salivary transforming growth factor beta (TGF-β), interleukin (IL)-35, and IL-17A. Correlations were not adjusted for confounding factors. A study of pregnant (*n* = 59), post-partum (*n* = 47), and healthy women (*n* = 70) also examined crude correlations between concentrations of salivary 25(OH)D and prostaglandin E_2_ and tumor necrosis factor-α (TNF-α) [18]. Differently, they observed a positive correlation between 25(OH)D and TNF-α in pregnant and post-partum women. Again, CRP was not assessed. There is evidence from in vitro studies that human gingival tissue can synthesize CRP [47] and we know it can be measured in saliva. A previous pilot study conducted a randomized controlled trial of either 4000 IU/day of vitamin D supplementation (*n* = 8) or placebo (*n* = 7) for 12 weeks. They observed a decrease in proinflammatory salivary biomarkers (TNF-α, IL-1β, IL-5, IL-6, and IL-10), but not chemokine ligand 20, epidermal fatty acid-binding protein, granulocyte-macrophage colony-stimulating factor, IL-2, IL-4, and IL-8 with vitamin D supplementation [19]. This study may indicate that vitamin D supplementation has some influence on local markers of oral inflammation, although it needs repeating in a more robust sample as the two arms were significantly different with respect to sex and smoking status. The results of our findings align with the results of a recent randomized, blinded, placebo-controlled clinical trial in 85 pregnant women in Pakistan [20]. They observed no changes in salivary cytokine profiles (IL-2, IL-4, IL-6, IL-10, TNF-α, interferon-γ, or IL-17) with supplementation of 4000 IU/day of vitamin D for 6 months.

Although the %CV for both serum and salivary CRP for our sample are considered acceptable for biomarkers (e.g., <25% [32]), serum CRP was at the higher range of acceptability (serum = 20.7%; saliva = 15.4% as reported [31]). Higher %CV increases the likelihood of loss of precision, and as such, the potential for random measurement error. Although it is unlikely that random measurement error is responsible for the null results of serum CRP, we cannot rule out its complete absence. Few participants in our study had detectable serum CRP concentrations > 10 mg/L, indicating that our study sample may not include participants presenting with acute inflammation or infection. It is possible that studies in those with more acute inflammation may observe different associations.

Participants in the OsteoPerio Study were predominantly non-Hispanic White, well-educated, postmenopausal women in overall good health. This may limit the external validity of our study findings to other populations. However, we suspect that the underlying biological mechanisms involved in vitamin D metabolism and the immune response should not vary (or minimally vary) by sex, race, and education level. Therefore, these results may still be generalized to some extent to other populations.

Despite some of these limitations, our study has a number of notable strengths. We were able to use biomarker measures of vitamin D status and oral and systemic inflammation, limiting potential measurement error. We were also able to adjust for a number of potential confounders, inclusive of dental hygiene measures, and explore the role of adiposity in this association using DXA measurements, which more accurately reflect adiposity in postmenopausal women compared to BMI measures [35]. Lastly, our study is novel and fills an important gap in the literature, shedding light on the potential relationship between vitamin D status and oral and systemic inflammation. In summary, circulating plasma 25(OH)D is not associated with systemic serum CRP or a marker of oral inflammation, as determined by concentrations of salivary CRP.

## Figures and Tables

**Table 1:** Characteristics (mean ± SD and n (%)) * of women in the OsteoPerio Study with measurements of plasma 25(OH)D according to quartiles (*n* = 567).

Demographic, Lifestyle, or Health Outcome		Quartile of Plasma 25(OH)D (nmol/L)			
	Overall Sample (*n* = 567)	Q1 (*n* = 142)	Q2 (*n* = 142)	Q3 (*n* = 141)	Q4 (*n* = 142)
Plasma 25(OH)D (nmol/L)	58.8 ± 22.6	31.3 ± 8.5	51.5 ± 5.0	64.9 ± 3.6	87.7 ± 15.5
Age (years)	66.8 ± 7.1	67.0 ± 7.4	67.5 ± 7.0	67.0 ± 7.0	65.7 ± 6.9
Race (% non-Hispanic White)	556 (98.1)	136 (95.8)	139 (97.9)	140 (99.3)	141 (99.3)
Healthy Eating Index—2015 ^†^	67.4 ± 10.0	64.4 ± 10.6	69.1 ± 10.1	68.3 ± 9.6	67.7 ± 9.0
Neighborhood Socioeconomic Status Index ^†^	76.3 ± 6.5	75.5 ± 7.0	76.9 ± 5.5	75.8 ± 6.5	76.9 ± 7.0
Smoking					
Never/Former	547 (96.5)	130 (91.6)	140 (99.3)	140 (99.3)	136 (95.8)
Current	20 (3.5)	12 (8.5)	1 (0.7)	1 (0.7)	6 (4.2)
Percent Body Fat	36.8 ± 5.9	38.9 ± 5.7	37.7 ± 5.5	36.0 ± 5.5	34.7 ± 6.1
Physical Activity (MET h/week) ^¶^					
≤1	103 (18.5)	39 (28.1)	23 (16.4)	20 (14.2)	21 (15.3)
>1 and <12.5	225 (40.4)	62 (44.6)	68 (48.6)	49 (34.8)	46 (33.6)
≥12.5	229 (41.1)	38 (27.3)	49 (35.0)	72 (51.1)	70 (51.1)
Hormone Therapy Use					
Never/Former	305 (53.8)	96 (67.6)	82 (57.8)	67 (47.5)	60 (42.3)
Current	262 (46.2)	46 (32.4)	60 (42.3)	74 (52.5)	82 (57.8)
History of Diagnosed Diabetes (% yes)	27 (4.8)	8 (5.6)	13 (9.2)	3 (2.1)	3 (2.1)
Oral Health & Hygiene Characteristics					
Number of Teeth	23.1 ± 5.4	22.0 ± 6.3	23.3. ± 5.5	23.2 ± 5.4	24.0 ± 4.2
Frequency of Brushing					
≤Once a day	136 (24.0)	42 (29.6)	38 (26.8)	24 (17.0)	32 (22.5)
Twice a day	309 (54.5)	74 (52.1)	78 (54.9)	82 (58.2)	75 (52.8)
>Twice a day	122 (21.5)	26 (18.3)	26 (18.3)	35 (24.8)	35 (24.7)
Frequency of Flossing					
Not every week	108 (19.1)	31 (21.8)	29 (20.4)	19 (13.5)	29 (20.4)
Once a week	55 (9.7)	8 (5.6)	15 (10.6)	16 (11.4)	16 (11.3)
>once a week	164 (28.9)	48 (33.8)	38 (26.8)	44 (31.2)	34 (23.9)
Everyday	240 (42.3)	55 (38.7)	60 (42.3)	62 (44.0)	63 (44.4)
Frequency of Dental Visits					
>1× per year	427 (75.3)	97 (68.3)	107 (75.4)	109 (77.3)	114 (80.3)
Once a year	88 (15.5)	22 (15.5)	24 (16.9)	20 (14.2)	22 (15.5)
Only with a problem/never	52 (9.2)	23 (16.2)	11 (7.8)	12 (8.5)	6 (4.2)
ACH Defined Periodontal Disease					
None	134 (23.6)	35 (24.7)	33 (23.2)	31 (22.0)	35 (24.7)
Mild/moderate	286 (50.4)	68 (47.9)	84 (59.2)	71 (50.4)	63 (44.4)
Severe	147 (25.9)	39 (27.5)	25 (17.6)	39 (27.7)	44 (31.0)
CDC/AAP Defined Periodontal Disease ^†^					
None/Mild	113 (20.2)	22 (15.8)	26 (18.6)	33 (23.7)	32 (22.5)
Moderate	350 (62.5)	91 (65.5)	98 (70.0)	81 (58.3)	80 (56.3)
Severe	97 (17.3)	26 (18.7)	16 (11.4)	25 (18.0)	30 (21.1)
Whole-mouth mean Pocket Depth	2.2 ± 0.4	2.3 ± 0.4	2.3 ± 0.4	2.2 ± 0.4	2.2 ± 0.4
Proportion of Bleeding on Probing (%)	35.9 ± 23.5	40.8 ± 26.1	36.1 ± 25.0	33.4 ± 19.2	33.2 ± 22.4

* Continuous variables presented as mean ± SD, categorical as n (%); ^†^ Sample size does not add up to 567 due to missing data for this variable. ^¶^ MET: metabolic equivalents.

**Table 2:** Beta coefficients, percentage change, and 95% CIs in salivary and serum C-reactive protein (CRP) concentrations per 10 nmol/L of 25(OH)D concentrations among postmenopausal women (*n* = 567) in the OsteoPerio Study.

				95% CI	
	$\hat{\beta }$	SE	Percentage Change	Lower Limit	Upper Limit
Salivary CRP (ng/mL) ^†^					
Unadjusted Model	−0.068	0.030	−6.57	−11.85	−0.99
Model 1 ^‡^	−0.079	0.030	−7.56	−12.78	−2.03
Model 2 ^§^	−0.025	0.029	−2.48	−7.88	3.24
Serum CRP (ng/mL) ^†^					
Unadjusted Model	−0.024	0.024	−2.37	−6.76	2.22
Model 1 ^‡^	−0.037	0.023	−3.62	−7.94	0.89
Model 2 ^§^	0.017	0.022	1.66	−2.65	6.16

^†^ Variable log-transformed; ^‡^ Model adjusted for age, smoking, frequency of brushing, and frequency of flossing, hormone therapy use (and Current); ^§^ Model 1 further adjusted for percent body fat.

**Table 3:** Beta coefficients, percentage change, and 95% CIs in salivary and serum CRP concentrations per 10 nmol/L of 25(OH)D concentrations among postmenopausal women (*n* = 567), stratified by % Body Fat (BF).

					95% CI	
	*n*	$\hat{\beta }$	SE	Percentage Change	Lower Limit	Upper Limit
Salivary CRP (ng/mL) ^†,‡^						
% BF < 40%	404	−0.057	0.036	−5.56	−11.93	1.28
% BF ≥ 40%	163	−0.039	0.051	−3.82	−12.92	6.23
Serum CRP (ng/mL) ^†,‡^						
% BF < 40%	404	−0.0047	0.026	−0.47	−5.46	4.77
% BF ≥ 40%	163	−0.0158	0.046	−1.57	−10.03	7.69

^†^ Variable log-transformed; ^‡^ Model adjusted for age, smoking, frequency of brushing, and frequency of flossing, hormone therapy use (Never/Former and Current).

## Data Availability

Data supporting the reported results can be found in data sets stored at the University at Buffalo in the research groups of Millen and Wactawski-Wende as well as data available through the Women’s Health Initiative (https://www.whi.org/, accessed on 29 March 2021).

## Supplementary Materials

The following are available online at https://www.mdpi.com/article/10.3390/nu13041148/s1, Figure S1: Study Sample Diagram, Figure S2: Scatterplot of the Association between Salivary and Serum CRP and Plasma 25(OH)D, Table S1: Characteristics (median [IQR] and n (%)) of women in the OsteoPerio Study with measurements of serum & salivary CRP according to tertiles (n = 567).

## Program Office

(National Heart, Lung, and Blood Institute, Bethesda, Maryland) Jacques Rossouw, Shari Ludlam, Joan McGowan, Leslie Ford, and Nancy Geller

## Clinical Coordinating Center

(Fred Hutchinson Cancer Research Center, Seattle, WA, USA) Garnet Anderson, Ross Prentice, Andrea LaCroix, and Charles Kooperberg

## Investigators and Academic Centers

(Brigham and Women’s Hospital, Harvard Medical School, Boston, MA, USA) JoAnn E. Manson; (MedStar Health Research Institute/Howard University, Washington, DC, USA) Barbara V. Howard; (Stanford Prevention Research Center, Stanford, CA, USA) Marcia L. Stefanick; (The Ohio State University, Columbus, OH, USA) Rebecca Jackson; (University of Arizona, Tucson/Phoenix, AZ, USA) Cynthia A. Thomson; (University at Buffalo, Buffalo, NY, USA) Jean Wactawski-Wende; (University of Florida, Gainesville/Jacksonville, FL, USA) Marian Limacher; (University of Iowa, Iowa City/Davenport, IA, USA) Jennifer Robinson; (University of Pittsburgh, Pittsburgh, PA, USA) Lewis Kuller; (Wake Forest University School of Medicine, Winston-Salem, NC, USA) Sally Shumaker; (University of Nevada, Reno, NV, USA) Robert Brunner.

## Women’s Health Initiative Memory Study

(Wake Forest University School of Medicine, Winston-Salem, NC, USA) Mark Espeland.
