# Supplementary material for: Plasma 25-Hydroxyvitamin D Concentrations and Serum and Salivary C-Reactive Protein in the Osteoporosis and Periodontal Disease Study
Source: Nutrients. 2021 Mar 31;13(4):1148. doi: 10.3390/nu13041148 (PMC8067277; doi:10.3390/nu13041148)
Supplement: Supplementary file 1 [file nutrients-13-01148-s001.pdf]

Supplemental Figure 1. Study Sample Diagram

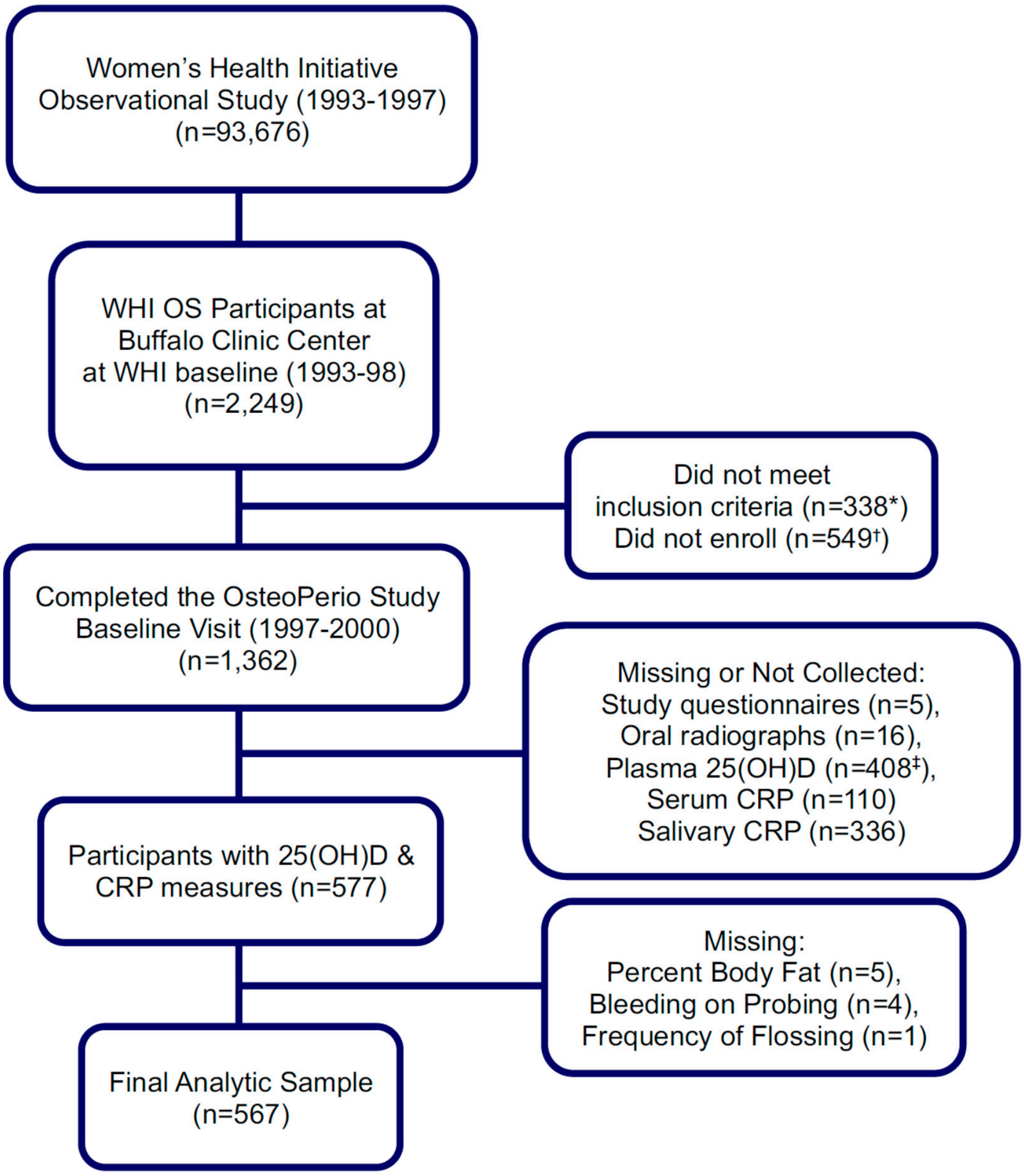

* Not eligible because they had fewer than 6 teeth (n=162), a history of bone disease (n=2), hip replacement (n=16), cancer diagnosed in last 10 years (n=106), or serious illness (n=52).
† Not included because they were unable to be reached (n=115), not interested (n=343), deceased (n=52), canceled after accepting (n=27), temporarily ineligible and unable to enroll before study end (n=12).
‡ Blood collection was added to the study protocol after the study initiation, therefore, stored plasma for assays of 25(OH)D were available for 934 of the 1,341 women enrolled. One woman had plasma 25(OH)D concentrations but was excluded because her value was 530 nmol/L.

Supplemental Figure 2a & b: Scatterplot of the Association between Salivary and Serum CRP and Plasma 25(OH)D

(a)

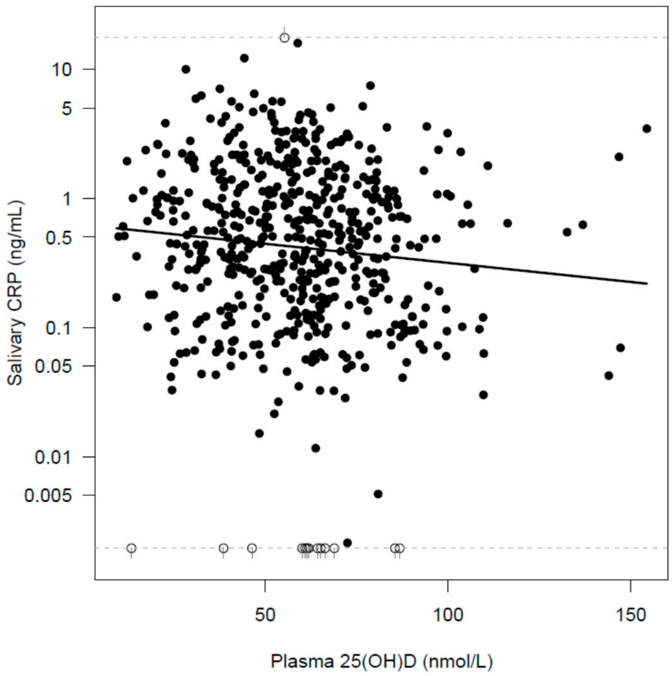

(b)

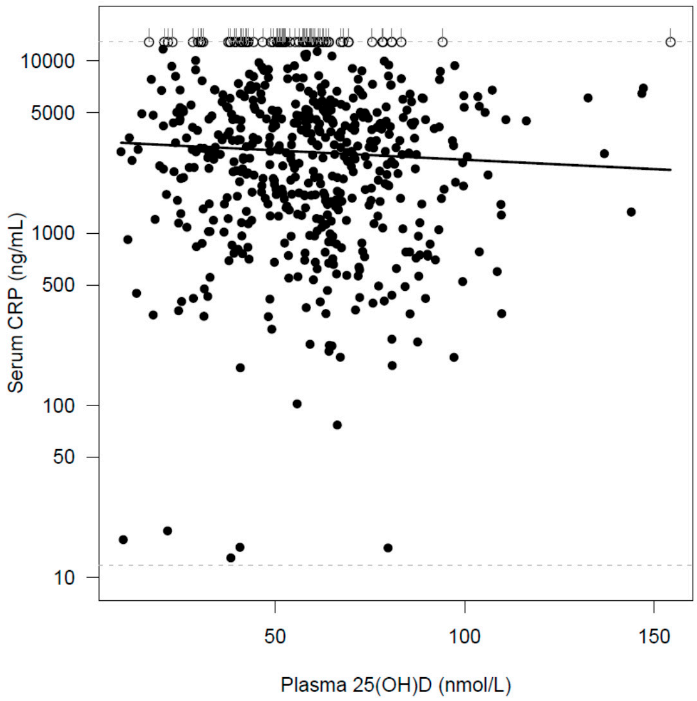

Legend: The scatterplot of salivary CRP and plasma 25(OH)D is shown in panel 2a and the scatterplot of serum CRP and plasma 25(OH)D is shown in panel 2b. For both 2a and 2b, the observed data is represented by filled circles. Data with CRP out of measurement range represented by open circles and line segments at limits of detection (dashed lines). CRP axis uses logarithmic scale.

Supplemental Table 1. Characteristics (median [IQR] and n (%)) of women in the OsteoPerio Study with measurements of serum & salivary CRP according to tertiles (*n*=567)								
**Demographic, Life Style, or Health Outcome**		**Salivary CRP (ng/mL)**				**Serum CRP (ng/mL)**		
	**Overall (*n=567*)**	**T1 (*n=189*)**	**T2 (n=*189*)**	**T3 (*n=189*)**	**Overall (*n=567*)**	**T1 (*n=188*)**	**T2 (n=*190*)**	**T3 (*n=189*)**
**CRP (ng/mL)† ‡**	0.51 (1.05)	0.11 (0.10)	0 .49 (0.32)	1.91 (1.68)	2,833.3 (3516.9)	959.7 (905.6)	3,260.7 (1470.4)	6,427.3 (2427.7)
**Healthy Eating Index-2015**	71.1 (14.4)	72.7 (13.9)	70.9 (13.4)	69.5 (16.6)		71.3 (16.7)	72.7 (14.1)	69.5 (15.8)
**Age (years)**	66.7 (11.4)	65.3 (12.1)	66.5 (11.4)	67.0 (11.1)		66.1 (11.4)	67.2 (12.0)	65.8 (11.3)
**Race (% non-Hispanic White)**	556 (98.1)	183 (96.8)	188 (99.5)	185 (97.9)		186 (98.4)	186 (98.4)	184 (97.4)
**Neighborhood Socioeconomic Status Index†**	77.7 (6.0)	77.4 (6.5)	78.2 (4.3)	77.43 (5.4)		77.7 (6.5)	77.9 (5.3)	77.6 (5.2)
**Smoking**								
Never/Former	547 (96.5)	184 (97.4)	182 (96.3)	181 (95.8)		181 (95.8)	184 (97.4)	182 (96.3)
Current	20 (3.5)	5 (2.7)	7 (3.7)	8 (4.2)		8 (4.2)	5 (2.6)	7 (3.7)
**Percent Body Fat**	37.5 (7.1)	34.4 (7.7)	37.7 (5.5)	39.1 (6.6)		34.8 (8.9)	37.6 (6.1)	38.6 (6.7)
**Physical Activity (MET hrs/wk)**								
≤ 1	192 (34.5)	56 (29.8)	63 (34.2)	73 (39.5)		54 (28.9)	65 (35.5)	73 (39.0)
> 1 and < 12.5	182 (32.7)	61 (32.4)	64 (34.8)	57 (30.8)		67 (35.8)	57 (31.1)	58 (31.0)
≥ 12.5	183 (32.9)	71 (37.8)	57 (31.0)	55 (29.7)		66 (35.3)	61 (33.3)	56 (29.9)

Supplemental Table 1. Characteristics (median [IQR] and n (%)) of women in the OsteoPerio Study with measurements of serum & salivary CRP according to tertiles (*n*=567)								
**Demographic, Life Style, or Health Outcome**	**Salivary CRP (ng/mL)**				**Serum CRP (ng/mL)**			
	**Overall (*n=567*)**	**T1 (*n=189*)**	**T2 (n=*189*)**	**T3 (*n=189*)**	**Overall (*n=567*)**	**T1 (*n=188*)**	**T2 (n=*190*)**	**T3 (*n=189*)**
**Hormone Therapy Use**								
Never/Former	305 (53.8)	125 (66.1)	100 (52.9)	80 (42.3)		120 (63.5)	100 (52.9)	85 (45.0)
Current	262 (46.2)	64 (33.9)	89 (47.1)	109 (57.7)		69 (36.5)	89 (47.1)	104 (55.0)
**History of Diagnosed Diabetes (% yes)**	27 (4.8)	5 (2.6)	6 (3.2)	16 (8.5)		3 (1.6)	9 (4.8)	15 (7.9)
**Number of Teeth**	25 (6)	25 (4)	25 (5)	24 (7)		26 (4)	25 (5)	24 (6)
**Frequency of Brushing**								
≤ Once a day	136 (24.0)	36 (19.0)	47 (24.9)	53 (28.0)		36 (19.0)	40 (21.2)	60 (31.7)
Twice a day	309 (54.5)	109 (57.7)	98 (51.9)	102 (54.0)		100 (52.9)	111 (58.7)	98 (51.9)
>Twice a day	122 (21.5)	44 (23.3)	44 (23.3)	34 (18.0)		53 (28.0)	38 (20.1)	31 (16.4)
**Frequency of Flossing**								
Not every week	108 (19.0)	39 (20.6)	34 (18.0)	35 (18.5)		37 (19.6)	28 (14.8)	43 (22.8)
Once a week	55 (9.7)	18 (9.5)	18 (9.5)	19 (10.1)		15 (7.9)	23 (12.2)	17 (9.0)
> once a week	164 (28.9)	46 (24.3)	58 (30.7)	60 (31.7)		44 (23.3)	60 (31.7)	60 (31.7)
Everyday	240 (42.3)	86 (45.5)	79 (41.8)	75 (39.7)		93 (49.2)	78 (41.3)	69 (36.5)
**Frequency of Dental Visits**								
> 1 x per year	427 (75.3)	145 (76.7)	153 (81.0)	129 (68.3)		151 (79.9)	149 (78.8)	127 (67.2)
Once a year	88 (15.5)	28 (14.8)	24 (12.7)	36 (19.0)		28 (14.8)	24 (12.7)	36 (19.0)

Supplemental Table 1. Characteristics (median [IQR] and n (%)) of women in the OsteoPerio Study with measurements of serum & salivary CRP according to tertiles (*n*=567)								
**Demographic, Life Style, or Health Outcome**	Salivary CRP (ng/mL)				Serum CRP (ng/mL)			
	Overall *(n=567)*	T1 *(n=189)*	T2 (n=*189)*	T3 *(n=189)*	Overall *(n=567)*	T1 *(n=188)*	T2 (n=*190)*	T3 *(n=189)*
Only with a problem/ never	52 (9.2)	16 (8.5)	12 (6.3)	24 (12.7)		10 (5.3)	16 (8.5)	26 (13.8)
**ACH Defined Periodontal Disease**								
None	134 (23.6)	46 (24.3)	48 (25.4)	40 (21.2)		39 (20.6)	48 (25.4)	47 (24.9)
Mild/moderate	286 (50.4)	102 (54.0)	93 (49.2)	91 (48.1)		106 (56.1)	87 (46.0)	93 (49.2)
Severe	147 (25.9)	41 (21.7)	48 (25.4)	58 (30.7)		44 (23.3)	54 (28.6)	49 (25.9)
**CDC/AAP Defined Periodontal Disease†**								
None/Mild	113 (19.9)	43 (22.8)	36 (19.0)	34 (18.0)		41 (21.7)	33 (17.5)	39 (20.6)
Moderate	350 (61.7)	117 (61.9)	117 (61.9)	116 (61.4)		120 (63.5)	120 (63.5)	110 (58.2)
Severe	97 (17.1)	25 (13.2)	35 (18.5)	37 (19.6)		25 (13.2)	35 (18.5)	37 (19.6)
**Whole-mouth mean Pocket depth**	2.2 (0.5)	2.2 (0.5)	2.23 (0.4)	2.2 (0.5)		2.1 (0.5)	2.2 (0.5)	2.2 (0.5)
**Proportion of Bleeding on Probing**	0.32 (0.33)	0.30 (0.31)	0.33 (0.36)	0.33 (0.35)		0.28 (0.32)	0.33 (0.32)	0.35 (0.37)

† Sample size does not add up to 567 due to missing data for this variable.

‡ CRP values within limit of detection presented (Salivary n=553, Serum n=501)
